# Time-related predictors for adverse events in polytrauma patients undergoing stand-alone definitive surgery

**DOI:** 10.1016/j.jcot.2025.103017

**Published:** 2025-04-16

**Authors:** Philipp Vetter, Cédric Niggli, Jan Hambrecht, Hans-Christoph Pape, Ladislav Mica

**Affiliations:** Department of Trauma Surgery, University Hospital Zurich, 8091, Zurich, Switzerland

**Keywords:** Multiple trauma, Sepsis, General surgery

## Abstract

**Background:**

Timely triage is crucial for polytrauma patients. Those with lower injury severity and physiological stress typically undergo isolated definitive surgery, but predictors for adverse events (AEs) in this group remain unclear. This study aims to identify time-related predictors of AEs in polytrauma patients undergoing stand-alone definitive surgery, excluding damage control interventions.

**Methods:**

We analyzed a trauma database spanning from 1996 to 2022, including 3653 patients. The focus was on individuals aged ≥16 years with an Injury Severity Score (ISS) ≥16 who underwent definitive orthopedic surgery. Injury and physiological parameters were recorded at admission and on the first and second days post-admission. Documented AEs included systemic inflammatory response syndrome (SIRS), sepsis, and mortality.

**Results:**

Among the 276 patients (mean age: 45.0 years with confidence interval, CI, 42.7–47.2 years; 71.7 % male; median ISS: 27 with interquartile range: 20–34), the incidence of SIRS was 79 % (n = 218), sepsis 13.8 % (n = 38), and mortality 4 % (n = 11). Upon admission, severe head and facial injuries and elevated leucocyte count (LC) predicted SIRS. Predictors for sepsis included ISS, heart rate, pH, and prothrombin time (PT), while non-survivors were older, with more severe head injuries and lower base excess (BE). On day one, elevated lactate levels were noted in both septic patients and non-survivors; LC predicted sepsis. By day two, higher lactate persisted in both groups, with non-survivors also showing reduced BE and PT. Primary (admission day) and multiple surgeries correlated with SIRS, whereas delayed surgeries were associated with sepsis. No surgical factors were correlated with mortality.

**Conclusion:**

Injury severity, physiological and surgical factors are associated with AEs in polytrauma patients undergoing definitive surgery. These findings with re-evaluation may help guide decision-making to minimize the risk of AEs.

**Level of evidence:**

Cohort-study, Level of Evidence = III.

**Trial registration:**

No. StV: 1-2008.

**Table:** List of abbreviations

AEs	= Adverse Events
AIS	= Abbreviated Injury Score
BE	= Base Excess
BMI	= Body Mass Index
CRP	= C-reactive Protein
GCS	= Glasgow Coma Scale
HB	= Hemoglobin
HR	= Heart Rate
ISS	= Injury Severity Score
LC	= Leucocyte Count
MAP	= Mean Arterial Pressure
PT	= Prothrombin Time
SBP	= Systolic Blood Pressure
SIRS	= Systemic Inflammatory Response Syndrome
TBI	= Traumatic brain injury
TC	= Thrombocyte Count

## 1 Introduction

Polytrauma remains a leading cause of mortality worldwide.^1^

Early triage based on injury^2^, ^3^, ^4^ and physiological parameters^2^^,^^4^, ^5^, ^6^, ^7^ is crucial to assess trauma-induced somatic stress, often referred to as the "first hit".^4^^,^^8^

This initial impact can trigger a systemic inflammatory response (SIRS) or, due to a compensatory anti-inflammatory response,^9^ lead to sepsis or mortality. These potential adverse events (AEs) must be considered in treatment planning to minimize morbidity, mortality and associated costs.

In polytrauma patients, multiple surgical interventions are often necessary. The timing (early vs. delayed) and approach (damage control vs. definitive) of these interventions can be adjusted unless urgent survival takes precedence. This “second hit” from surgery^2^^,^^4^ aims to optimize early mobility^10^ while managing the procedural stress of surgery.^3^^,^^8^

Patients with lower injury severity^3^^,^^4^ and reduced physiological stress may be eligible for isolated definitive orthopedic surgery without damage-control procedures.^4^^,^^10^ However, determining the optimal timing, based on physiological parameters, remains a subject of debate.^11^

This study explores time-related predictors of AEs in polytrauma patients undergoing isolated definitive surgery, with the goal of identifying patients who may qualify for early surgical intervention.

## 2 Methods

### 2.1 Patient sample

This retrospective cohort study reviewed our institutional trauma database (University Hospital of Zurich), which includes 3653 patients between 1996 and 2022. Inclusion criteria for patients were age ≥16 years, an ISS ≥16, and stand-alone definitive orthopedic trauma surgery, meaning that cases with any orthopedic damage control procedures (such as external fixation) were excluded.

Injury factors (trauma type, accident type, ISS,^12^ Abbreviated Injury Score^13^) and physiological markers were recorded at admission and reassessed on days one and two after admission [i.e., Glasgow Coma Scale (GCS), body temperature, body mass index, heart rate (HR), systolic blood pressure, mean arterial pressure, pH, Base Excess (BE), lactate, C-reactive protein (CRP), hemoglobin (HB), thrombocyte count (TC), prothrombin time (PT), and leucocyte count (LC)]. Surgical parameters, including the type and timing (primary: on admission day, secondary: thereafter) of procedures, were documented, along with AEs (SIRS, sepsis, mortality) within 21 days post-admission. AEs had to occur within 21 days of observation.

### 2.2 Definition of sepsis

Sepsis was defined as a SIRS score^14^ of 2 or more, with an identified infection source.^15^^,^^16^ The diagnosis of an infection was made by either based on strong clinical criteria (especially low blood pressure, signs of organ dysfunction, or apparent hypoperfusion) or by the finding of a positive microbiological culture.

### 2.3 Laboratory and statistical analysis

All laboratory analyses were conducted using standardized equipment at a single institution (University Hospital of Zurich), with acid-base parameters measured in Point-of-Care Testing (blood gas analysis).

### 2.4 Statistics

Data were analyzed using SPSS 29.0. A Chi-square test, Mood's median test, and Mann-Whitney *U* Test (as there was no normal distribution according to the Shapiro-Wilk-Test) were used to assess intergroup differences for categorical, ordinal, and metric variables. Binary logistic regression served for evaluation of predictive parameters for AEs. In this regard, if significant factors measured on admission day were identified, then the subsequent analysis for day one and two was corrected for these factors by inclusion into the regression analysis. Statistical significance was set at p < 0.05.

### 2.5 Ethical approval

Ethical approval was granted by the ethics commission of the University Hospital of Zurich and the local government of Zurich upon database implementation (Nr. StV: 1–2008), later reapproved for (BASEC 2021-00391). The study adheres to good clinical practice, the Declaration of Helsinki, and the TRIPOD guidelines.^17^

## 3 Results

The cohort of 276 patients (Table 1) exhibited SIRS in 79 % (n = 218), sepsis in 13.8 % (n = 38), and mortality in 4 % (n = 11) of cases.

**Table 1:** Patient sample characteristics at admission.

Parameter at admission	n	Value
Age	276	45.0 (42.7–47.2)^a^
Sex (male)	276	71.7 (198)^c^
Blunt trauma	276	97.1 (268) c
Type of accident	276	
- Work		13.0 (36) c
- Traffic		55.0 (152) c
- Sports/Leisure		9.4 (26) c
- At home		10.9 (30) c
- Suicide		4.7 (13) c
- Delinquency		0.7 (2) c
- Other		6.2 (17) c
ISS	276	27 (20–34) b
AIS (0–5)	276	
- Head	276	26/12/8/24/14/16 (71/33/21/67/40/44)^c^
- Face	276	66/7/19/8/1/0 (181/20/51/22/2/0^c^
- Thorax	276	35/3/5/40/14/3 (96/8/15/111/37/9)^c^
- Abdomen	276	67/2/8/11/9/3 (185/4/21/31/26/9)^c^
- Pelvis	276	68/1/10/17/4/0 (187/3/27/48/11/0^c^
- Spine	276	48/0/13/26/5/8 (133/0/37/71/14/21)^c^
- Extremities	276	23/5/28/34/9/1 (63/14/79/93/25/2)^c^
- Integument	276	63/26/10/1/0/0 (174/73/28/1/0/0)^c^
GCS	270	14 (7–15) b
Body temperature	200	35.9 (35.7–36.0) a
BMI	197	24.2 (22.3–26.2)^b^
HR	204	89 (87–92) a
SBP	203	132 (129–135) a
MAP	168	95 (92–97) a
pH	197	7.34 (7.33–7.36) a
BE	214	−2.36 (−2.80 to −1.91) a
Lactate	237	2.17 (2.00–2.33) a
CRP	226	21.6 (14.5–28.7) a
HB	246	12.1 (11.0–13.1) a
TC	237	218 (207–229) a
PT	230	87 (75–100) b
LC	263	13.4 (12.8–14.1) a

Age [years]; AIS, Abbreviated Injury Score; BE, Base Excess [mmol/L]; BMI, Body Mass Index [kg/m2]; CRP, C-reactive Protein [mg/L]; GCS, Glasgow Coma Scale; HR, Heart Rate [per minute]; HB, Hemoglobin [g/dL]; ISS, Injury Severity Score; Lactate [mmol/L]; LC, Leucocyte Count [G/L]; MAP, Mean Arterial Pressure [mmHg]; PT, Prothrombin Time [% of reagence]; SBP Systolic Blood Pressure [mmHg]; SIRS, Systemic Inflammatory Response Syndrome; Body Temperature [°C]; TC, Thrombocyte Count [G/L].

a Mean with 95 % confidence interval.

b Median with interquartile ranges.

c Percentages with number.

Sepsis sources included trachea-bronchial fluid (n = 22), bacteremia (n = 7), catheter infections (n = 3), wound, urinary tract and cerebrospinal fluid infections (n = 2 each). Causes of mortality were traumatic brain injury (TBI; n = 6), cardiopulmonary failure (n = 2), sepsis-related cardiovascular failure (n = 1), and cardiac arrest (n = 1).

Predictors and group differences associated with the development of SIRS are reported in Table 2, while the corresponding analysis regarding sepsis development and mortality are depicted in Table 3, Table 4, respectively.

**Table 2:** Group comparison of admission parameters according to SIRS development.

SIRS						
Parameter at admission	n	Value -No SIRS-	n	Value -SIRS-	p-value	Prediction for SIRS (BLR)
AIS (0–5)						
- Head	58	21/17/5/24/21/12 (12/10/3/14/12/7)^c^	218	27/11/8/24/13/17 (59/23/18/53/28/37)^c^	0.342	**0.029**
- Face	58	54/10/24/10/2/0 (31/6/14/6/1/0)^c^	218	69/6/17/7/1/0 (150/14/37/16/1/0)^c^	0.246	**0.009**
- Extremities	58	22/3/45/21/9/0 (13/2/26/12/5/0)^c^	218	23/6/24/37/9/1 (50/12/53/81/20/2)^c^	**0.045**	0.255
GCS	57	15 (14–15) b	213	14 (2–15)^b^	**<0.001**	0.609
Body temperature	35	36.4 (36.1–36.7)^a^	165	35.8 (35.6–36.0)^a^	**0.008**	0.199
pH	37	7.37 (7.34–7.40)^a^	160	7.34 (7.33–7.35)^a^	**0.005**	0.341
BE	41	−1.51 (−2.11 to −0.91)^a^	173	−2.56 (−3.09 to −2.02)^a^	**0.035**	0.514
CRP	46	25.7 (10.1–41.2)^a^	180	20.6 (12.5–28.6)^a^	**0.042**	0.613
HB	50	14.5 (9.5–19.5)^a^	196	11.4 (11.1–11.8)^a^	**0.044**	0.426
PT	50	94 (80–109)^b^	180	86 (57–115)^b^	**<0.001**	0.127
LC	56	12.4 (10.9–13.9)^a^	207	13.7 (13.0–14.5)^a^	**0.016**	**0.04**

AIS, Abbreviated Injury Score; BE, Base Excess [mmol/L]; BLR, Binary logistic regression; CRP, C-reactive Protein [mg/L]; GCS, Glasgow Coma Scale; HB, Hemoglobin [g/dL]; Lactate [mmol/L]; LC, Leucocyte Count [G/L]; PT, Prothrombin Time [% of reagence]; SIRS, Systemic Inflammatory Response Syndrome; Body Temperature [°C].

a Mean with 95 % confidence interval.

b Median with interquartile ranges.

c Percentages with number.

**Table 3:** Group comparison of admission parameters according to sepsis development.

Sepsis						
Parameter at admission	n	Value -No Sepsis-	n	Value -Sepsis-	p-value	Prediction for Sepsis (BLR)
Sex (male)	238	69.3 % (165)^c^	38	86.8 % (33)^c^	**0.026**	0.066
ISS	238	26 (12–40)^b^	38	35 (20–50)^b^	**<0.001**	**0.026**
AIS (0–5)						
- Head	238	24/11/8/27/15/15 (58/25/20/65/36/34)^c^	38	34/21/3/5/11/26 (13/8/1/2/4/10)^c^	**0.008**	0.235
- Thorax	238	37/3/6/37/15/2 (89/8/14/88/34/5)^c^	38	18/0/3/61/8/10 (7/0/1/23/3/4)^c^	**0.003**	0.339
HR	177	88 (86–91)^a^	27	98 (90–107)^a^	**0.018**	**0.008**
pH	170	7.35 (7.34–7.36)^a^	27	7.31 (7.28–7.35)^a^	**0.034**	**0.016**
PT	198	87 (63–111)^b^	32	84 (39–130)^b^	0.680	**0.003**

AIS, Abbreviated Injury Score; BLR, Binary logistic regression; HR, Heart Rate [per minute]; ISS, Injury Severity Score; PT, Prothrombin Time [% of reagence].

a Mean with 95 % confidence interval.

b Median with interquartile ranges.

c Percentages with number.

**Table 4:** Group comparison of admission parameters according to mortality.

Mortality						
Parameter at admission:	n	Value -No mortality-	n	Value -Mortality-	p-value	Prediction for mortality (BLR)
Age	265	44.1 (41.9–46.4)^a^	11	65.0 (54.0–76.1)^a^	**<0.001**	0.064
Type of accident						
- Suicide	–	4.2 (11)^b^	–	18.2 (2)^b^	**0.031**	0.883
AIS						
- Head	265	26/12/9/25/14/14 (70/33/21/65/38/38)^b^	11	9/0/0/18/18/55 (1/0/0/2/2/6^b^	**0.013**	0.577
BE	206	−2.26 (−2.71 to −1.80)^a^	8	−4.91 (−7.26 to −2.57)^a^	**0.017**	0.647

AIS, Abbreviated Injury Score; BE, Base Excess [mmol/L]; BLR, Binary logistic regression.

^2^: median with interquartile ranges.

median with interquartile ranges.

a Mean with 95 % confidence interval.

b Percentages (with number).

On day one, there were still group differences and predictors for sepsis development (Table 5).

**Table 5:** Parameters at day 1 (day after admission).

	Sepsis						Mortality					
Parameter at day 1	n	Value -No Sepsis-	n	Value -Sepsis-	p-value	Sepsis (BLR)	n	Value -No mortality-	n	Value -Mortality-	p-value	Mortality (BLR)
Lactate	182	1.09 (0.99–1.18)^a^	30	1.50 (1.26–1.74)^a^	**<0.001**	0.692	208	1.12 (1.04–1.21)^a^	8	2.25 (1.14–3.36)^a^	**0.003**	0.995
TC	158	168 (155–182)^a^	24	138 (112–165)^a^	**0.042**	0.071	177	165 (152–178)^a^	8	129 (91–167)^a^	0.179	0.999
PT	150	88 (68–108)^b^	22	83 (54–112)^b^	**0.035**	0.746	167	88 (68–108)^b^	8	81 (44–118)^b^	0.543	0.999
LC	188	9.2 (8.7–9.7)^a^	27	10.2 (8.4–12.1)^a^	0.321	**0.047**	211	9.2 (8.8–9.8)^a^	8	10.3 (5.6–15.0)^a^	0.649	0.996

All cases with past sepsis or mortality are excluded for the respective analysis. BLR, Binary logistic regression; Lactate [mmol/L]; LC, Leucocyte Count [G/L]; PT, Prothrombin Time [% of reagence]; TC, Thrombocyte Count [G/L].

a Mean with 95 % confidence interval.

b Median with interquartile ranges.

On day two, only group differences related to sepsis and mortality were noted, but no respective predictors (Table 6).

**Table 6:** Parameters at day 2 (second day after admission).

	Sepsis						Mortality					
Parameter at day 2	n	Value -No Sepsis-	n	Value -Sepsis-	p-value	Sepsis (BLR)	n	Value -No mortality-	n	Value -Mortality-	p-value	Mortality (BLR)
BE	93	1.20 (0.59–1.81)^a^	20	1.10 (−0.11-2.30)^a^	0.406	0.296	111	1.39 (0.89–1.89)^a^	6	−2.42 (−6.59-1.76)^a^	**0.012**	0.997
Lactate	110	1.07 (0.95–1.20)^a^	21	1.32 (1.11–1.54)^a^	**0.005**	0.569	128	1.07 (0.97–1.18)^a^	6	2.62 (1.59–3.65)^a^	**<0.001**	0.990
PT	113	93 (78–108)^b^	21	88 (62–114)^b^	0.334	0.949	132	93 (77–109)^b^	5	70 (44–96)^b^	**0.033**	0.981

All cases with past sepsis or mortality are excluded for the respective analysis. BE, Base Excess [mmol/L]; BLR, Binary logistic regression; Lactate [mmol/L]; PT, Prothrombin Time [% of reagence].

a Mean with 95 % confidence interval.

b Median with interquartile ranges.

Surgical predictors were found for both SIRS and sepsis, but not for mortality (Table 7).

**Table 7:** Surgical parameters predicting SIRS, sepsis and mortality.

	SIRS	Sepsis	Mortality	Percentage of total cohort (n)
Sum of primary surgeries	**0.025 (r=0.806)**	0.143	0.996	1: 41 % (113) 2: 5 % (15) 3: 1 % (3)
Sum of total (orthopedic) surgeries	**0.03** **(r=0.635)**	0.577	0.996	1: 69 % (189) 2: 25 % (70) 3: 4 % (11) 4: 2 % (6)
Day of secondary spine surgery	0.714	**0.005 (r=0.271)**	0.642	Total: 28 % (78): 1: 8 % (21) 2: 7 % (20) 3 and later: 13 % (37)
Day of forearm plating surgery	0.364	**0.014 (r=0.312)**	1	Total: 22 % (60): 0: 4 % (12) 1: <1 % (2) 2: 2 % (n = 5) 3 and after: 15 % (n = 41)
Day of femoral nailing surgery	0.998	**0.05 (r=0.695)**	0.998	Total: 15 % (41) 0: 13 % (36) 1: <1 % (1) 3 and after: 1 % (4)
Day of femoral screws surgery	0.998	**0.04 (r=0.403)**	0.998	Total: 17 % (46) 0: 15 % (42) 1: <1 % (2) 3 and after: <1 % (2)

Significant p-values are presented with the exponentiation coefficient. If procedures are reported by respective day, then their sum is described first. SIRS, Systemic Inflammatory Response Syndrome.

Among concomitant non-orthopedic procedures [ventricle probes (n = 16), subdural space probes (n = 14), chest tubes (n = 30) and thoracotomies (n = 68)], only thoracotomies were linked to sepsis (r = 0.492; p = 0.01).

## 4 Discussion

This study examined the association between injury severity, physiological markers, and surgical interventions with AEs in polytrauma patients undergoing isolated definitive orthopedic surgery. The analysis yielded several key findings:

• **At Admission:** Significant differences in injury and physiological markers were identified as predictors for SIRS, sepsis, and mortality.
• **Post-Admission Days 1 and 2:** These predictive differences persisted, particularly with elevated lactate levels.
• **Surgical Insights:** An increased number of surgical procedures was correlated with SIRS, while delayed treatment of extremity injuries was associated with sepsis development. Mortality, however, did not correlate with surgical factors.

Efficient triaging and intervention in the trauma bay are critical, highlighting injury severity and physiological stability as key criteria for decision-making.^2^, ^3^, ^4^

While various studies have evaluated the timing and types of treatment in polytrauma management,^2^, ^3^, ^4^^,^^10^^,^^18^ comprehensive predictive models remain scarce, especially for less critically injured patients.^3^

### 4.1 Comparative findings

This study presents a framework consistent with established criteria for determining the feasibility of definitive surgery over damage-control strategies.^18^^,^^19^ Fröhlich et al.^19^ analyzed 31.154 patients (mean age: 45 years; 73 % male, mean ISS: 28) and reported a sepsis rate of 10.4 %, slightly lower than ours, while their mortality rates were higher (6.2 % 30-day mortality, 16.5 % in-hospital mortality). Although their primary outcome focused on multi-organ failure, many of the risk factors identified—age, male sex, ISS, head and thorax injury severity, BE, and coagulopathy—align with those observed in our cohort. Additionally, shock, coagulopathy, hypothermia, and acidosis are well-documented key indicators in polytrauma management.^2^, ^3^, ^4^, ^5^, ^6^, ^7^^,^^20^, ^21^, ^22^, ^23^

### 4.2 Injury severity and physiological stress

Age and injury severity are critical, unalterable factors influencing complications and outcomes.^2^ The ISS was found to correlate with sepsis in our study (specific group difference of ISS 35 vs. 26 for sepsis development), and age was significantly associated with mortality (with no 95 % CI overlap at 50 years between survivors and non-survivors).

Head injuries (AIS 5), particularly TBI,^2^^,^^6^ highlighted the importance of injury profile. TBI can delay surgical timing due to the need for initial neurosurgical procedures, prolonged monitoring, and risk of impaired cerebral perfusion due to blood loss.^8^ Extended immobilization from delayed treatment or invasive ventilation could also increase the risk of respiratory infections.

### 4.3 Physiological parameters and adverse events

Acid-base indicators (pH, BE, lactate) demonstrated relative stability and resuscitation effectiveness^4^ in our cohort, However, even slight deviations from normal values (pH < 7.35; BE < -2 mmol/L; lactate >2 mmol/L)^4^^,^^5^^,^^21^^,^^22^ were associated with all three AEs, underscoring the relevance of these factors in predicting AEs.^2^^,^^4^^,^^6^^,^^7^^,^^20^^,^^21^

Regarding coagulopathy, PT emerged as a factor for sepsis at both the early (admission day) and late (second day after admission) time point, confirming its role in polytrauma surgery.^23^ However, PT was not particularly specific, as the only significant group difference in PT was observed on day one (84 vs. 87 % of reagence).

### 4.4 Surgical interventions and timing

Increased surgical procedures performed on the admission day correlated with SIRS, suggesting a surgery-dependent physiological response,^2^^,^^24^ but not with sepsis. Delayed treatment of fractures was linked to a higher risk of sepsis, in line with findings by Kobbe et al.^25^ who reported that early fixation reduced septic complications, and shortened Intensive-care-unit length of stay, promoting early mobilization and rehabilitation.^10^

### 4.5 Limitations

This study has several limitations. Comorbidities, particularly renal diseases affecting acid-base balance,^26^ were not considered. Changes in resuscitation strategies over time (with now less aggressive resuscitation), more complex antibiotic treatment and evolving surgical technique could have introduced variability. There is also institutional bias, as our level 1 trauma center's focus on severe TBI cases. Furthermore, the low mortality rate might have limited the ability to identify statistically significant results. The results only describe correlations and not causations. However, they can provide valuable insights into markers that should be considered at different time points, potentially improving re-assessment and resource allocation.

Larger, prospective studies with narrower time intervals are required to provide more precise data on AEs in polytrauma patients undergoing stand-alone definitive surgery.

## 5 Conclusion

Injury severity, physiological and surgical factors are associated with AEs in polytrauma patients undergoing definitive surgery. These findings with re-evaluation may help guide decision-making to minimize the risk of AEs.

## CRediT authorship contribution statement

**Philipp Vetter:** Data curation, Formal analysis, Investigation, Software, Visualization, Writing – original draft. **Cédric Niggli:** Data curation, Project administration, Software, Writing – review & editing. **Jan Hambrecht:** Data curation, Project administration, Software, Writing – review & editing. **Hans-Christoph Pape:** Data curation, Project administration, Software, Writing – review & editing. **Ladislav Mica:** Conceptualization, Data curation, Investigation, Methodology, Project administration, Resources, Supervision, Validation, Writing – review & editing.

## Ethical approval

Ethical approval was granted by the ethics commission of the University Hospital of Zurich and the local government of Zurich upon database implementation (Nr. StV: 1–2008), later reapproved for (BASEC 2021-00391). The study was performed in accordance with the ethical standards as laid down in the 1964 Declaration of Helsinki and its later amendments or comparable ethical standards.

## Consent for publication

Not applicable.

## Availability of data and materials

Production data is available upon reasonable request.

## Funding

IBM WATSON Health Pathway Explorer: Innovation Funding INOV00040 and INOV00092; University Hospital Zurich; 8091 Zurich; Switzerland; 06/2017 and 09/2019; Ladislav Mica. Copyrights of WATSON Health Pathway Explorer by Ladislav Mica and IBM.

## Declaration of competing interest

The authors declare that they have no known competing financial interests or personal relationships that could have appeared to influence the work reported in this paper.
